# Merging of Accidental Bound States in the Continuum in Symmetry and Symmetry-Broken Terahertz Photonic Crystal Slabs

**DOI:** 10.3390/nano15060451

**Published:** 2025-03-16

**Authors:** Jiale Chen, Jianjun Liu, Fangzhou Shu, Yong Du, Zhi Hong

**Affiliations:** Centre for THz Research, China Jiliang University, Hangzhou 310018, China; S22030810003@cjlu.edu.cn (J.C.); jianjun@cjlu.edu.cn (J.L.); fzshu@cjlu.edu.cn (F.S.); yongdu@cjlu.edu.cn (Y.D.)

**Keywords:** bound states in the continuum, photonic crystal slab, guided mode resonance

## Abstract

Recently, the merging of accidental bound states in the continuum (BICs) has attracted significant attention due to the enhanced light–matter interactions. Here, we theoretically demonstrate the merging of accidental BICs in perturbed all-silicon terahertz photonic crystal (PhC) slabs with *C*_2_ and *C*_2_ broken-symmetry structures. The PhC slabs consist of an array of four cylindrical holes and support a TM symmetry protected (SP) vector BIC at the Γ point. Our results indicate that the merging and band transition of accidental BICs can be achieved by varying the diameter of diagonal holes in a *C*_2_-symmetry structure. Notably, in a *C*_2_ broken-symmetry PhC slab, the SP BIC will first convert to a quasi-BIC, then transit to a new accidental BIC, which are well displayed and interpreted by tracing the accidental BICs in momentum space. We believe that the results presented in this work show potential for the design and application of BICs in both symmetric and asymmetric PhC slabs.

## 1. Introduction

Bound states in the continuum (BICs) have attracted intensive attention due to the enhanced light–matter interactions [1,2,3,4] and have been widely applied in optical nonlinearity [5,6], low-threshold lasers [7,8], and high-sensitivity sensing [9,10]. Photonic crystal (PhC) slabs serve as an effective platform for the generation of both symmetry-protected (SP) BICs [6,11,12,13] and accidental BICs [14,15,16]. The nonradiative features of BICs in PhC slabs can be attributed to their topological far-field polarization characteristics in momentum space, where BICs are identified as vortices carrying integer topological charges [17,18]. These BICs are also referred to as topologically protected vector BICs or topological defects [13,19,20,21,22]. Because of the conservation of topological charges, the accidental BICs can be easily tuned in momentum space by varying the structural parameters, such as the dielectric constant, lattice constant, thickness, or other geometric parameters, while preserving the symmetry of the structure [21,22,23,24,25,26]. Thus, various merged BICs in PhC slabs, such as accidental BICs merges with SP BIC at Γ point, accidental BIC merges with other accidental BIC, or guided mode resonance (GMR) at off-Γ points, can be achieved. By merging multiple topological charges, not only radiative losses in a large wave vector range, but also out-of-plane scattering losses caused by fabrication imperfections or disorders can be strongly suppressed, resulting in a greatly enhanced Q-factor of the resonance. Experimental results have demonstrated a merged BIC with a Q value of up to 10^6^ in optic regime [27,28], enhanced laser outputs [29,30,31], and high-sensitivity sensing [32].

Recently, Lee et al. demonstrated that by adjusting the thickness of PhC slabs, the merged accidental BICs at the Γ point will band transition to a related GMR, resulting in a merged BIC within the GMR band [33,34]. Additionally, merged BICs can be achieved in perturbed PhC slabs by changing the geometric parameters [35]. However, currently, lots of related studies focus on PhC slabs with *C*_4_ symmetry, while there is a scarcity of research on PhC slabs with *C*_2_ symmetry, and no reports on *C*_2_ symmetry-broken PhC slabs. In addition, the underlying physics of numerous accidental BICs in asymmetric PhC slabs or metasurfaces remains unclear [26,36,37].

Here, we theoretically investigate the merging and band transition of accidental BICs in perturbed all-silicon PhC slabs with *C*_2_ and *C*_2_ broken-symmetry structures. The PhC slabs consist of an array of four cylindrical holes in a high-resistivity silicon substrate and operate at terahertz (THz) frequencies. The results demonstrate that the merging and band transition of accidental BICs can be easily manipulated by altering the diameters of diagonal holes of the structure. The evolution of accidental BICs in momentum space clearly indicates that accidental BICs will firstly merge at two off-Γ points, then move along the Γ–M direction and merge with a SP BIC at the Γ point, thereafter band transitioning to a related GMR, which is different from the evolution observed in *C*_4_ symmetry PhC slabs. Notably, in a *C*_2_ symmetry-broken PhC slab, as the diameter of one hole varies, the SP BIC will first convert to a quasi-SP BIC, then transit to a new accidental BIC, which can be interpreted as the result of destructive interference of two quasi-accidental BICs and the quasi-SP BIC. Furthermore, a leaky GMR can become a BIC just as occurred in the *C*_2_-symmetry PhC slab. Therefore, the results presented in this work give a new way to interpret the formation of BICs in both symmetric and asymmetric PhC slabs.

## 2. Result and Discussion

### 2.1. Accidental BICs in a Perturbed PhC Slab with C_4_ Symmetry

**Figure 1:**
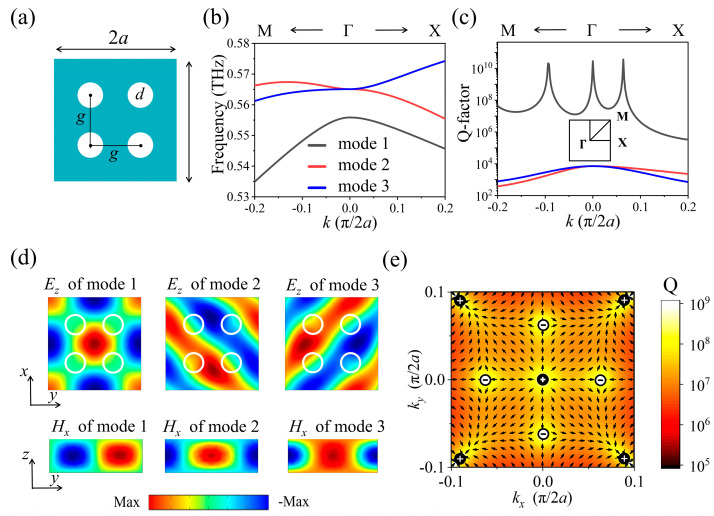
(**a**) Schematic diagram of a perturbed all-silicon PhC slab consisting of an array of four cylindrical air holes. The periods of the PhC slab along the *x*-axis and *y*-axis are *a_x_* = *a_y_* = 2*a* = 300 μm. (**b**,**c**) Dispersion curves and Q-factors of three TM eigenmodes along the M–Γ–X direction, when *d* = 62.5 μm and *g* = 120 μm. The inset illustrates the first Brillouin zone of the square lattice. (**d**) Electric and magnetic field distributions of three modes at the Γ point, where white circles represent the holes. (**e**) Q-factor and polarization field distribution of mode 1 in momentum space. There are nine *k* points, whose Q-factors approach infinity (highlight), i.e., BICs with different topological charges of +1 and −1.

We begin our investigation with a *C*_4_-symmetry all-silicon terahertz PhC slab consisting of an array of four cylindrical air holes. As illustrated in Figure 1a, the periods of the PhC slab along the *x*-axis and *y*-axis are *a_x_* = *a_y_* = 2*a* = 300 μm. The diameters of the four holes are equal and marked as *d*, and the distance between the centers of two adjacent holes in both the *x* and *y* directions is *g*. The thickness of the PhC slab is *t* = 120 μm. When *g* is not equal to *a*, a period-perturbation is introduced into this PhC slab. In the following, *g* is fixed at 120 μm for the perturbed PhC slabs. Such a kind of PhC slab working in the THz band is easy for fabrication [38,39]. The finite element method (COMSOL Multiphysics 5.6) was utilized to conduct the eigenmode analysis of the perturbed PhC slab. In calculations, period boundary conditions were employed in both the *x* and *y* directions, along with a perfectly matched layer (PML) and a 500 μm-thick air layer between the PML and the top of the PhC slab in the *z* direction. In addition, perfect electric conductors (PECs) in both the PML layer and the bottom of PhC slab with a half thickness of 60 μm were used for transverse magnetic (TM) eigenmode calculations, while the finer meshes were chosen, and lossless silicon with a dielectric constant of 11.67 was assumed.

Dispersion curves and Q-factors calculated from the complex eigenfrequency (Re/2Im) for two considered TM eigenmodes are calculated within the frequency range of 0.53 to 0.58 THz when *d* = 62.5 μm and presented in Figure 1b,c. The two modes are designated as mode 1 and mode 2. At the Γ point, the frequencies of mode 1 and mode 2 are 0.556 THz and 0.565 THz, respectively, and their electric field distributions (*E_z_*) in the *x*–*y* plane are illustrated in Figure 1d. According to the propagation of the wavefront, and considering the infinite Q values of mode 2 in the whole wave vector range when *g* = *a* and finite Q values when *g* ≠ *a*, shown in Figure 1c, we can get that mode 2 is a GMR excited by (1, 1) Rayleigh diffraction of a two-dimensional metagrating with a period of 2*a* in the *x* and *y* directions [38,40]. Actually, there is another, last mode, mode 3, in the considered frequency range, as shown in Figure 1b–e. Mode 3 is also a GMR, which is degenerated with mode 2 at the Γ point and propagates in a –45° direction with respect to the *x*-axis, while it is +45° for mode 2. In fact, when *g* = *a*, mode 1, mode 2, and mode 3 are the modes that lie below the light cone and are forbidden to leak out due to total internal reflections (TIRs). By introducing period-perturbation (when *g* ≠ *a*), the periods of the PhC slab in both the *x* and *y* directions can be doubled to 2*a*; thus, the Brillouin zone folding occurs, allowing access to these modes from free-space excitation and transitioning these non-radiative dark modes into radiative resonances [40]. In addition, mode 1 and mode 2, mode 1 and mode 3 are two pair of modes corresponding to asymmetric and symmetric solutions (*H*_x_, as shown in Figure 1d), respectively [41,42]. The Q-factor of mode 1 approaches infinity at the Γ point and drops rapidly when *k* deviates from the Γ point. Therefore, mode 1 at the Γ point is a typical SP BIC. In contrast, mode 2 and mode 3 are leaky GMRs. Notably, when *k* further deviates from the Γ point along the Γ–M and Γ–X directions, the Q-factor of mode 1 exhibits two resonant peaks, reaching infinity at *k* = −0.094 and *k* = 0.064, which are referred to as accidental BICs.

In fact, according to the *C*_4_ symmetry of the PhC slab, there are eight accidental BICs within the momentum space, as illustrated in Figure 1e. Furthermore, mode 1 will appear as singularities of far-field polarization vectors in momentum space, acting as vortex centers that carry conserved and quantized topological charges. The topological charge (*q*) carried by BICs is defined as follows [17]:

(1) $$q=\frac{1}{2\pi }\oint_{C}dk\cdot {▽}_{k}\phi \left(k\right),q\in Z$$

where ϕ(k) represents the angle between the polarization vector and the *x*-axis, and *C* refers to a closed trajectory within momentum space that travels around the BICs in the counterclockwise direction. The overall angle of the polarization vector undergoes a change of +2π (−2π) after completing a counterclockwise loop, resulting in a topological charge (*q*) of +1 (−1). As shown in Figure 1e, the calculated topological charges are +1 for the four accidental BICs, and −1 for the other four accidental BICs, while the topological charge of SP BIC is +1. According to the conservation of topological charge, accidental BICs can be sustained within momentum space as structural parameters vary, provided that the symmetry is maintained [33]. Therefore, we can manipulate the eight accidental BICs of mode 1 within momentum space to move toward the Γ point, and merge with the SP BIC or transition to mode 2 or mode 3 by adjusting the thickness of the PhC slab. These processes can also be achieved by varying the structure parameters *d* and *g*. However, currently, lots of studies focus on PhC slabs with *C*_4_ symmetry, whereas there are few works on PhC slabs with *C*_2_ symmetry. In the following, we will concentrate our investigation on the perturbed PhC slabs with *C*_2_ symmetry and even with *C*_2_ broken symmetry.

### 2.2. Merging and Band Transition of Accidental BICs in a PhC Slab with C_2_ Symmetry

**Figure 2:**
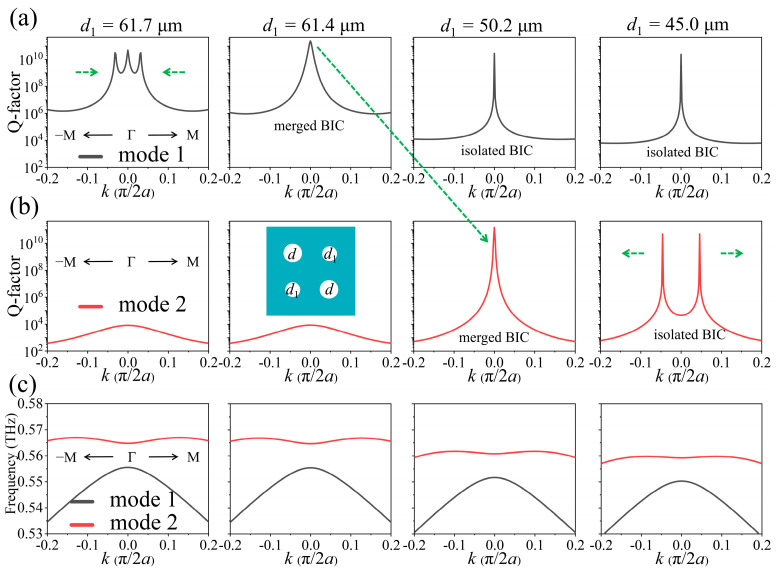
(**a**,**b**) Evolution of radiative Q-factors of mode 1 and mode 2 with *d*_1_ for a perturbed *C*_2_-symmetry PhC slab where *d* = 62.5 μm. Inset is a structure diagram of the PhC slab, where the diameters of the diagonal holes are marked as *d* and *d*_1_, respectively. As *d*_1_ decreases, multiple accidental BICs firstly merged with a SP BIC at the Γ point, then the merged BICs transition from mode 1 to mode 2, while the SP BIC consistently appears at mode 1. (**c**) Dispersion curves at different *d*_1_.

A perturbed PhC slab with *C*_2_ symmetry is illustrated in the inset of Figure 2, where the diameters of the diagonal holes are labeled as *d* and *d*_1_, respectively. The evolution of the radiative Q-factors for mode 1 and mode 2 with respect to the diameter *d*_1_ are calculated and displayed, respectively, in Figure 2a,b, and dispersion curves in Figure 2c where *d* = 62.5 μm. When *d*_1_ = 61.7 μm, three isolated resonant peaks (*k* is within the range of −0.20 to 0.20) appear on the Q-factor curve of mode 1. These peaks can be attributed to two accidental BICs located at *k* = ±0.032 and one SP BIC at the Γ point. As *d*_1_ decreases from 61.7 μm, the two accidental BICs move toward the Γ point along the Γ–M and merge with the SP BIC at the Γ point when *d*_1_ = 61.4 μm, resulting in a single enhanced peak in the Q-factor curve of mode 1. In addition, compared to the isolated SP BIC or accidental BIC, the merged BIC exhibits significantly improved radiative Q-factors over a wide range of wavevectors. As *d*_1_ further decreases to 50.2 μm, the merged accidental BICs will transition from mode 1 to mode 2, making mode 2 from a leaky GMR into a BIC with infinite Q-factors. As *d*_1_ continues to decrease, the merged accidental BICs in mode 2 will move away from the Γ point. Thus, the merging and band transition of accidental BICs can be easily manipulated in the perturbed PhC slabs with *C*_2_ symmetry.

**Figure 3:**
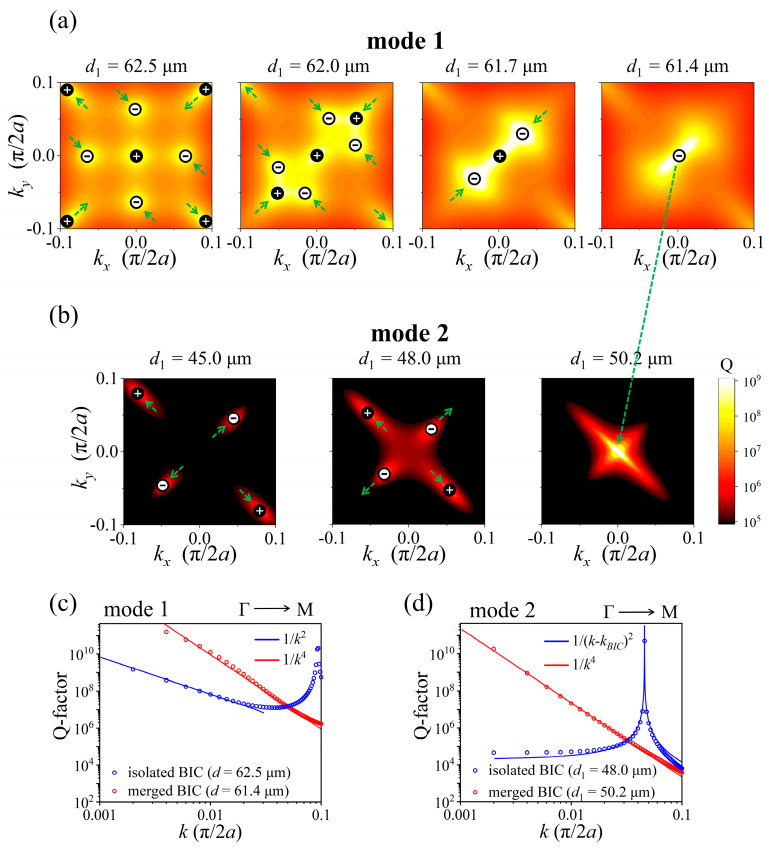
(**a**,**b**) Evolution of the merging and band transition of accidental BICs in momentum space when *d*_1_ decreases from 62.5 μm to 45.0 μm. Q-factor distributions for mode 1 and mode 2. Topological charges of ±1 indicate the locations of the BICs in momentum space; green arrows illustrate the movements of the accidental BICs, showing that multiple BICs with topological charges of ±1 can merge at both the Γ point and off-Γ points. (**c**,**d**) Q-factors for merged BICs and isolated BICs in PhC slabs with different *d*_1_ for mode 1 and mode 2. Red and blue circles represent merged BICs and isolated BICs, respectively, while solid lines indicate their fittings.

In order to more intuitively and deeply understand the merging and band transition of accidental BICs, we investigate the evolution of accidental BICs in momentum space by varying *d*_1_, as illustrated in Figure 3a,b. As *d*_1_ decreases from 62.5 μm to 61.7 μm, four accidental BICs with −1 topological charges simultaneously detach from the Γ–X and Γ–Y directions and subsequently move to the Γ–M direction at a specific angle of 135°. Meanwhile, among the four accidental BICs with +1 topological charges, two gradually move along the Γ–M direction and approach the Γ point, while the other two move away from it. We can clearly see that all eight accidental BICs and an SP BIC are protected when the structure maintains *C*_2_ symmetry, whereas they are tuned in momentum space along the two mirror symmetry axes at ±45° directions with respect to the *x*-axis. Specifically, some accidental BICs move along one symmetry axis towards or away from another one, while other BICs move towards or away from the Γ point. As a result, when *d*_1_ = 61.7 μm, the Q-factor at the two positions of (0.032, 0.032) and (−0.032, −0.032) in momentum space exhibits significant enhancement along the Γ–M direction. This phenomenon occurs because at each position, the two accidental BICs with −1 topological charges move to the Γ–M direction and then merge with an accidental BIC with +1 topological charge that is approaching the Γ point. As a result, two merged BICs with −1 topological charges are formed (See polarization distributions in Figure S1 in Supplementary Materials), whereas this will not happen in *C*_4_ symmetry PhC slabs. When *d*_1_ further decreases to 61.4 μm, the two merged BICs with −1 topological charges will continue to move toward the Γ point, ultimately merging with the SP BIC at the Γ point, leading to a significantly enhanced Q-factor in the Γ–M direction near the vicinity of the Γ point, and it is evident that the merged BIC has a topological charge of −1, as shown in Figure S1. Interestingly, as *d*_1_ continues to decrease from 61.4 μm, an isolated SP BIC remains at the Γ point, but has a topological charge of −1, as shown in Figure S1. This means that the topological charge of the SP BIC switches from +1 to −1 at this point. In addition, when *d*_1_ = 50.2 μm, the Q-factor of mode 2 at the Γ point becomes infinite, which is a result from the band transition of the merged accidental BICs from mode 1 to mode 2. And as *d*_1_ further decreases, the four isolated accidental BICs at mode 2 move away from the Γ point; among them, the two with +1 topological charges move along the Γ–M direction at −45°, while the two with −1 topological charges move along Γ–M. The polarization distributions of mode 2 in momentum space are displayed in Figure S1.

**Figure 4:**
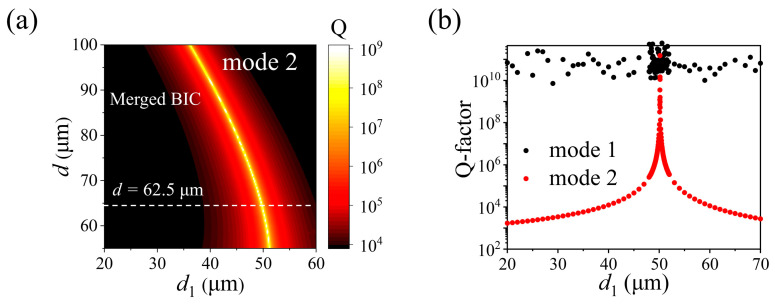
(**a**) Q-factor of mode 2 in (*d*, *d*_1_) geometric parameter space at the Γ point. The merged BIC is highlighted due to its infinite Q-factor. (**b**) Q-factors of mode 1 and mode 2 with respect to *d*_1_, where *d* = 62.5 μm.

Moreover, Q-factors of the merged BICs and isolated BICs in PhC slabs with different *d*_1_ for mode 1 and mode 2 are also extracted and plotted in Figure 3c,d. By fitting the Q-factor curves, we find that the Q-factor of the isolated BICs for mode 1 at *d*_1_ = 62.5 μm and mode 2 at *d*_1_ = 48.0 μm are all inversely proportional to the square of the wave vector *k* (Q ∝ *k*^−2^) [27], whereas Q ∝ *k*^−4^ for the Q-factor of the merged BICs for mode 1 at *d*_1_ = 61.4 μm and mode 2 at *d*_1_ = 50.2 μm, just as that for the merged BICs in *C*_2_-symmetry structures [23]. Furthermore, we have also simulated the Q-factor of mode 2 at the Γ point for the PhC slabs with varying *d* (from 55.0 μm to 100.0 μm) and *d*_1_ (from 20.0 μm to 60.0 μm), as illustrated in Figure 4a. In this figure, the band transition of the merged BICs is highlighted due to its infinite Q-factor, which means that the merged BICs can be easily manipulated by adjusting the diameters of the holes in PhC slabs with *C*_2_ symmetry. In addition, the extracted Q-factors for mode 1 and mode 2 with respect to *d*_1_, when *d* = 62.5 μm, are presented in Figure 4b. It is clear to see that the Q-factor of mode 1 at the Γ point remains infinite throughout the entire range of *d*_1_, signifying the presence of the SP BIC when the *C*_2_ symmetry of the PhC slab is maintained.

We should mention here that merging and band transition of accidental BICs can also happen between mode 1 and mode 3, as shown in Figure S2. For fixed *d*_1_ = 62.5 μm, as *d* decreases to 61.4 μm, the accidental BICs will merge with the SP BIC at the Γ point in mode 1, then band transition to mode 3 at *d* = 50.2 μm, making mode 3 become a BIC.

### 2.3. Accidental BICs in a PhC Slab with C_2_ Broken Symmetry

**Figure 5:**
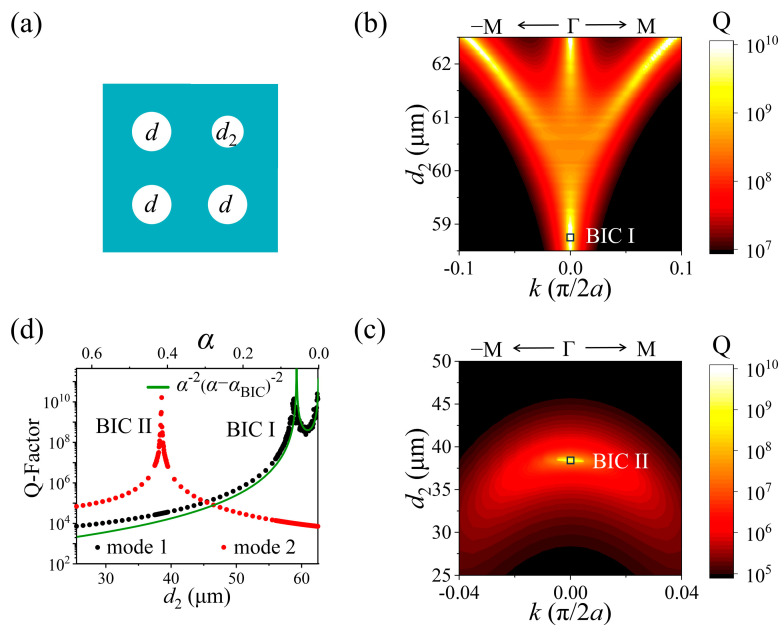
(**a**) Schematic diagram of a *C*_2_ symmetry-broken PhC slab. (**b**,**c**) Q-factor of mode 1 and Q-factor of mode 2 within the geometric parameter space of (*k*, *d*_2_), where *d* = 62.5 μm. (**d**) Q-factors of mode 1 and mode 2 at the Γ point with varying *d*_2_.

Finally, we shift our investigation to a *C*_2_ symmetry-broken PhC slab, as illustrated in Figure 5a. For simplicity, we take the diameter of the three holes to equal *d* = 62.5 μm, with *d*_2_ used for the one remaining. Although the *C*_2_ symmetry of the structure is broken, mirror symmetry along Γ–M is still retained. Q-factors of mode 1 and mode 2 within the geometric parameter space of (*k*, *d*_2_) are calculated and presented in Figure 5b and Figure 5c, respectively, and their extracted values at the Γ point varying with *d*_2_ are displayed in Figure 5d.

It is evident that, once the *C*_2_ symmetry of the PhC slab is broken (*d*_2_ ≠ *d*), the SP BIC of mode 1 at the Γ point will collapse into a quasi-SP BIC. Consequently, the Q-factor of mode 1 will decrease and follow Q ∝ *α*^−2^, where *α* = (*d* − *d*_2_)/*d* represents an asymmetric parameter of the structure [12]. Meanwhile, the eight accidental BICs are not topologically protected either, but converted into quasi-accidental BICs, which we here we call leaky defect modes. The trajectory of the Q-factor with *d*_2_ for mode 1 in the geometric parameter space shown in Figure 5b is quite the same as that with *d*_1_ in *C*_2_-symmetry PhC slab. As *d*_2_ decreases from 62.5 μm, three leaky defect modes will meet at the Γ–M direction, along with the other three at the Γ−M direction, then move towards the Γ point. They reach the Γ point when *d*_2_ = 60.2 μm and remain at the Γ point when *d*_2_ decreases further. Interestingly, we found that the Q-factor of mode 1 does not decrease as *d*_2_ decreases from 60.2 μm, but rises quickly and approaches infinity again when *d*_2_ = 58.8 μm, as shown in Figure 5d; that is, a new “accidental” BIC is formed (see polarization field distribution in Figure S3), labeled as BIC I, which is attributed to the destructive interference of the two leaky defect modes and the quasi-SP BIC. Hence, the Q-factor of mode 1 follows Q ∝ *α*^−2^ (*α*-*α*_BIC I_)^−2^, as shown in Figure 5d. Furthermore, as *d*_2_ decreases further, we can see in Figure 5c,d that there is a peak in the Q-factor curve of mode 2 when *d*_2_ = 38.5 μm; that is, the leaky GMR of mode 2 becomes a BIC (marked as BIC II). This BIC is similar to that observed in *C*_2_-symmetry PhC slabs; hence, it can be interpreted as the destructive interference of the two leaky defect modes band transitioned from mode 1 to mode 2.

**Figure 6:**
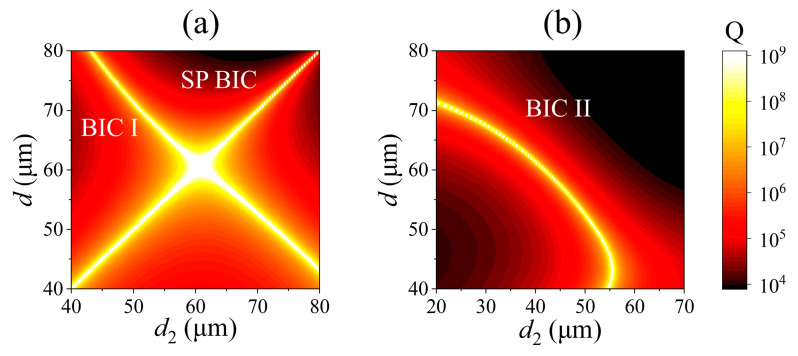
(**a**,**b**) Extracted Q-factor at the Γ point for mode 1 and mode 2 in the (*d*, *d*_2_) geometric parameter space.

In addition, Q-factors of mode 1 and mode 2 at the Γ point in the (*d*, *d*_2_) geometric parameter space are calculated and presented in Figure 6a and Figure 6b, respectively. Two distinct BICs, BIC I and SP BIC, are observed in mode 1. They are very close together at parameters (61 μm, 61 μm), and separate far apart with greater parameter deviation. Meanwhile, BIC II is clearly observed over a wide range of parameter variations, which is similar to that in a *C*_2_-symmetry PhC slab, shown in Figure 4.

## 3. Conclusions

In summary, we numerically demonstrate the merging and band transition of accidental BICs in perturbed all-silicon THz PhC slabs with *C*_2_ and *C*_2_ broken-symmetry structures. The PhC slab consists of an array of four cylindrical holes and supports a SP vector BIC at the Γ point. The merging and band transition of accidental BICs can be easily manipulated by changing the diameter of the diagonal holes of the structure. The evolution of accidental BICs with the diameter of holes in momentum space clearly shows that accidental BICs will firstly merge at two off-Γ points, then move along the Γ–M direction and merge with the SP BIC at the Γ point, and thereafter band transition to a related GMR, which is different from the evolution in *C*_4_-symmetry PhC slabs. Especially, in a *C*_2_ symmetry-broken PhC slab, via changing the diameter of one hole, the SP BIC will firstly convert to a quasi-BIC, then transit to a new accidental BIC, which is attributed to the destructive interference of two leaky defect modes and the quasi-SP BIC. Furthermore, a leaky GMR will become a BIC that results from the band transition of the leaky defect modes. The proposed method of tracing accidental BICs from symmetric to asymmetric structures in momentum space is greatly helpful for understanding the formation of BICs in PhC slabs. We believe that the PhC slabs presented in this work have great potential in terahertz sensing and other high Q related functional devices. The evolutions of accidental BICs in the proposed PhC slabs vs. the distance g or lattice constant a, as well as the merging of transverse electrical (TE) BICs, can be further studied in the future.

## Data Availability

The data are available on reasonable request from the corresponding author.

## Supplementary Materials

The following supporting information can be downloaded at https://www.mdpi.com/article/10.3390/nano15060451/s1, Figure S1: Polarization field distributions in momentum space for mode 1 and mode 2 in a perturbed *C*_2_-symmetry PhC slab; Figure S2: Comparison of merging and band transition of accidental BICs between mode 1 and mode 2 and between mode 1 and mode 3; Figure S3: Polarization field distribution in momentum space for BIC I with topological charge of −1 when *d*_2_ = 58.8 μm and BIC II when *d*_2_ = 38.5 μm.
