# Supplementary material for: Merging of Accidental Bound States in the Continuum in Symmetry and Symmetry-Broken Terahertz Photonic Crystal Slabs
Source: Nanomaterials (Basel). 2025 Mar 16;15(6):451. doi: 10.3390/nano15060451 (PMC11945727; doi:10.3390/nano15060451)
Supplement: Supplementary file 1 [file nanomaterials-15-00451-s001.zip › nanomaterials-3501411-supplementary.pdf]

Merging of accidental BICs in symmetry and symmetry-broken terahertz photonic crystal slabs

Jiale Chen, Jianjun Liu, Fangzhou Shu, Yong Du, and Zhi Hong*

1. Polarization field distributions in momentum space for mode 1 and mode 2 in a perturbed *C*2-symmetry PhC slab

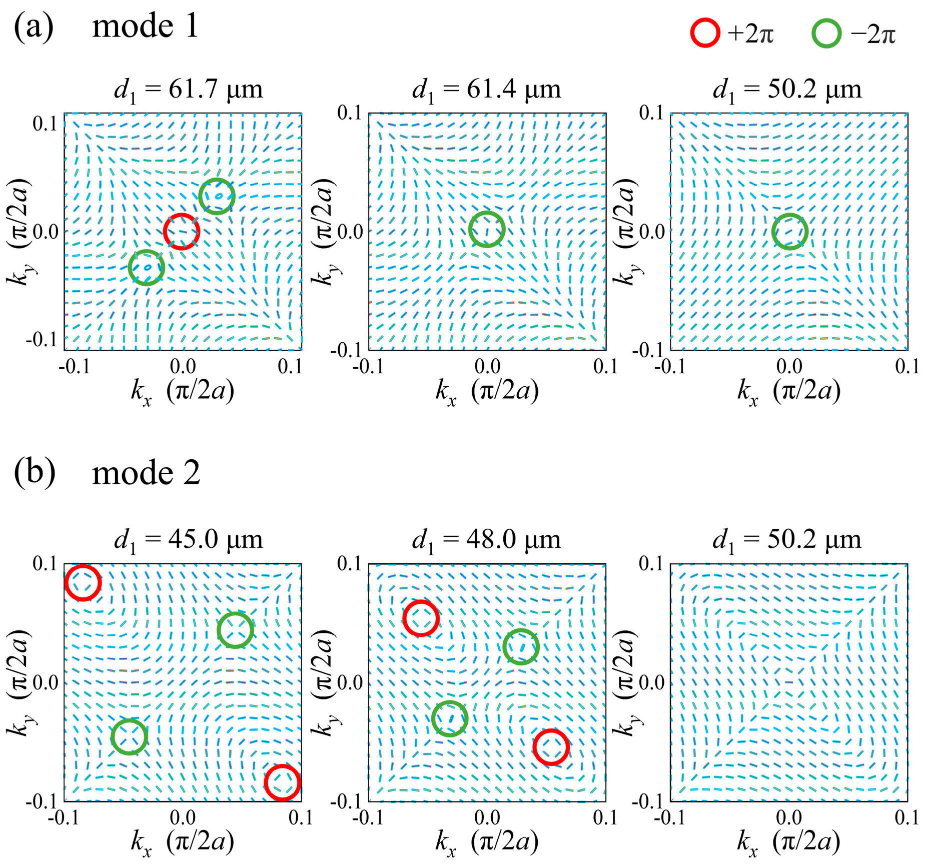

Figure S1. (**a,b**) Polarization field distributions in momentum space for mode 1 and mode 2 in a perturbed *C*2-symmetry PhC slab. The red (green) circle means the polarization vectors return to their original positions after completing a counterclockwise loop, resulting in a change in the overall angle of +2π (−2π); therefore, the topological charge (q) is +1 (−1).

2. Comparison of merging and band transition of accidental BICs between mode 1 and mode 2 and between mode 1 and mode 3.

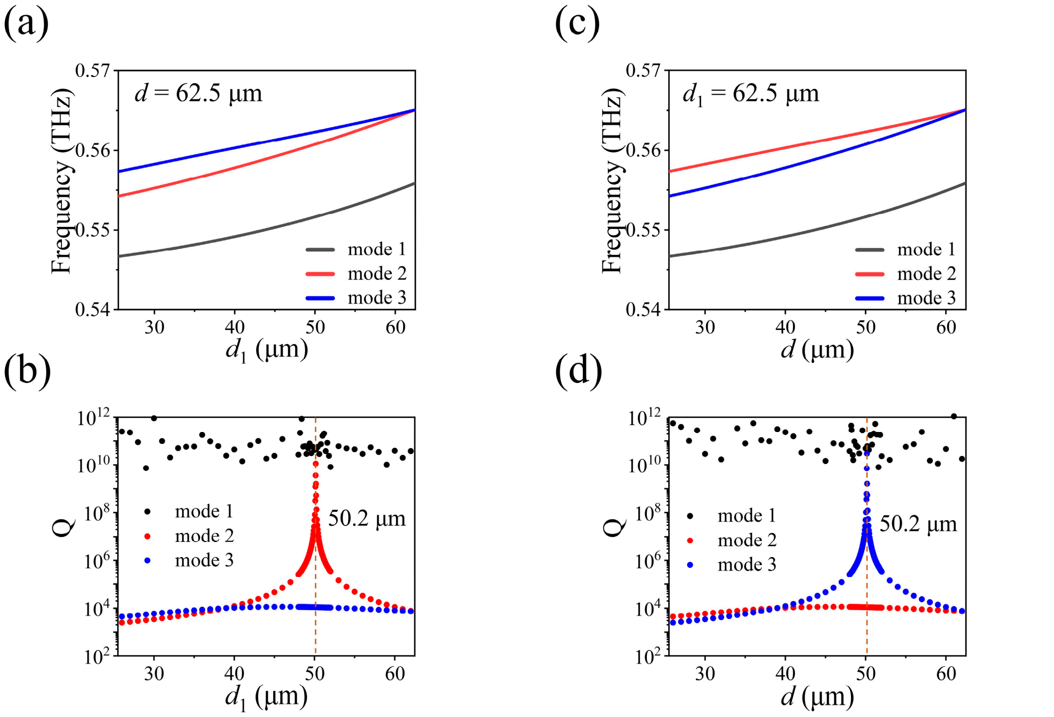

Figure S2. (**a,b**) Frequencies and Q-factors of three modes vs. d_1 when d = 62.5 μm. (**c,d**) Frequencies and Q-factors of three modes vs. d when d_1 = 62.5 μm.

3. Polarization field distribution in momentum space for BIC I and BIC II

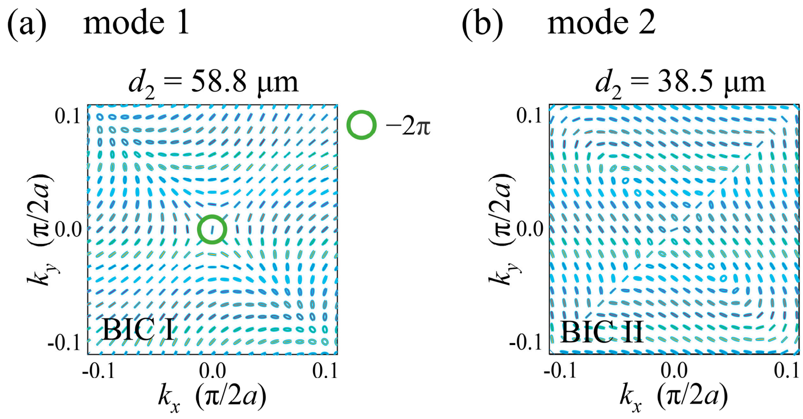

Figure S3. (**a,b**) Polarization field distribution in momentum space for BIC I with topological charge of −1 when d_2 = 58.8 μm and BIC II when d_2 = 38.5 μm.
